# Research and Clinical Practice Involving the Use of *Cannabis* Products, with Emphasis on Cannabidiol: A Narrative Review

**DOI:** 10.3390/ph17121644

**Published:** 2024-12-06

**Authors:** João Luís Q. Simei, José Diogo R. Souza, João Francisco Pedrazzi, Francisco S. Guimarães, Alline Cristina Campos, Antônio Zuardi, Jaime Eduardo C. Hallak, José Alexandre S. Crippa

**Affiliations:** 1 Department of Neuroscience and Behavior, Ribeirão Preto Medical School, University of São Paulo, Ribeirão Preto 14049-900, São Paulo, Brazil; joao.simei@usp.br (J.L.Q.S.); jose.diogo.souza@usp.br (J.D.R.S.); joaofranciscopedrazzi@usp.br (J.F.P.); awzuardi@fmrp.usp.br (A.Z.); jhallak@fmrp.usp.br (J.E.C.H.); 2 National Institute for Science and Technology-Translational Medicine, Ribeirão Preto 14049-900, São Paulo, Brazil; fsguimar@fmrp.usp.br; 3 Department of Pharmacology, Ribeirão Preto Medical School, University of São Paulo, Ribeirão Preto 14049-900, São Paulo, Brazil; allinecampos@usp.br

**Keywords:** cannabis, phytocannabinoids, cannabidiol, endocannabinoid system, pharmacology, drug development, medical cannabinoids, medical cannabis

## Abstract

Background: Emerging evidence supports cannabidiol (CBD) as a promising therapeutic compound for various health conditions, despite its approval as a medication (product for medical purposes) remaining restricted to a limited range of clinical indications. Simultaneously, the regulation of cannabis-derived products for medicinal and recreational use has expanded their global market availability to meet local community demands. This scenario presents a complex challenge for clinicians, researchers, and industry, as the global appeal of therapeutic uses of CBD is growing more rapidly than the scientific evidence supporting its safety and effectiveness. Outcomes: A narrative review was conducted to discuss the best evidence regarding the pharmacological profile of CBD, its efficacy, and safety within the context of regulation and perspectives on the development of new cannabinoid-based drugs. Key articles addressing the various facets of this issue were selected for comprehensive analysis. Conclusions: Clinicians and researchers may face unique challenges in understanding the pharmacological profile of CBD and the prospects for developing its clinical indications, given the heterogeneity of clinical terminologies and the quality and composition of cannabis-based medical products available on the market. More basic and clinical research that complies with regulatory agencies’ testing guidelines, such as good manufacturing practices (GMPs), good laboratory practices (GLPs), and good clinical practices (GCPs), is needed to obtain approval for CBD or any other cannabinoid as a therapeutic for broader clinical indications.

## 1. Introduction

In the past two decades, there has been growing interest in the therapeutic uses of cannabidiol (CBD), one of more than 129 phytocannabinoids found in the cannabis plant. In contrast with Δ-9-tetrahydrocannabinol (∆9-THC), the primary component responsible for the psychotomimetic effects associated with the plant, CBD lacks these properties and is not associated with the reinforcement properties that may lead to abuse or misuse [1,2,3]. CBD also has a favorable tolerance profile across a wide range of doses [4] and has not been associated with changes in critical physiological parameters such as blood pressure, heart rate, or body temperature [2,5], garnering attention for its therapeutic applications due to its low side-effects profile [3,6].

In parallel with academic discussion and research, social perspectives toward the cannabis plant have undergone significant shifts, resulting in notable changes in legislation, public policy, and the marketing of cannabis and its derivatives in many countries [7,8]. Since the advancement of regulatory frameworks and the growth of the cannabis-derived products market over the past decade [8], there has been a marked increase in the variability of cannabinoid-containing products intended for both recreational and medical use [9,10,11]. However, the boundaries between these products are sometimes blurred, as in the case of “over-the-counter” CBD products [12,13,14,15].

**Figure 1:**
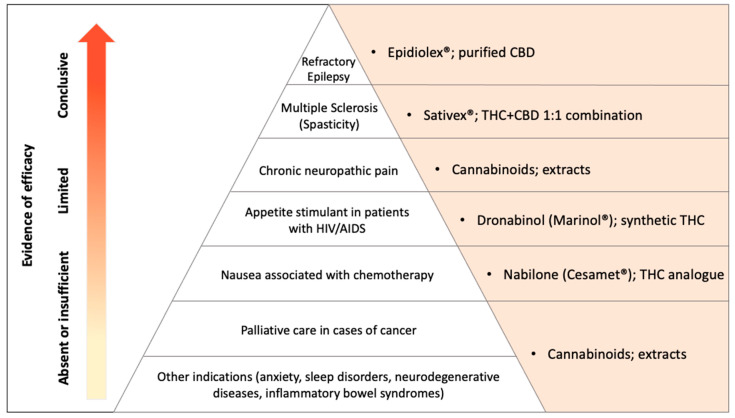
Therapeutic claims of phytocannabinoids and cannabis-derived products in the literature and their respective evidence of efficacy.

Within the scope of products intended for medical purposes under medical prescription, there is currently a wide variability, including isolated (e.g., Epidiolex^®^) or combined (e.g., nabiximols, Sativex^®^, Mevatyl^®^) phytocannabinoids of pharmacological grade purity, synthetic cannabinoids and their analogs (e.g., dronabinol and nabilone), CBD-enriched cannabis extracts that may not contain or contain traces (less than 0.3%) of THC (“broad spectrum”) or may contain THC in variable amounts (“full spectrum”), and the whole cannabis plant for medical use [16]. Although this variability can improve patient access [8], few of these products are pharmaceutical grade and have their applications supported by gold-standard evidence from randomized placebo-controlled clinical trials (RCTs) for the various indications they are currently used for, even when medically prescribed [17] (see Figure 1).

The only products that have demonstrated sufficient efficacy and safety for their approval as medications by regulatory agencies are Epidiolex^®^ for refractory childhood epilepsy in Lennox–Gastaut and Dravet syndromes and in tuberous sclerosis complex [18,19,20]. Also, Sativex^®^, a 1:1 combination of THC and CBD, was approved to control spasticity and neuropathic pain in multiple sclerosis [21,22]. Additionally, there is still weak evidence of the efficacy of Δ9-THC and analogs for the relief of chemotherapy-induced vomiting and nausea and appetite stimulation in HIV wasting syndrome [21,23], although the FDA has approved these indications [17].

The high variability of marketed products, while beneficial for improving patient access, presents significant challenges for clinical decision-making in prescribing due to the broad variability of regulatory hurdles, pharmaceutical best practices, and quality control (difficulty in standardizing market cannabis-derived products regarding the quantity, proportion, and stability of phytocannabinoids) [8,17,24]. This complexity arises not only from the challenges of handling the plant’s botanical matrix but also because these marketed products often do not adhere to good agricultural practices (GAPs) and good manufacturing practices (GMPs), nor are they subjected to adequate pharmacovigilance controls through good laboratory practices (GLPs) [24,25,26,27]. These issues have several crucial implications for clinical practice, most notably the lack of effectiveness and safety concerns [28]. The variable amounts of ∆9-THC [11,27] and associated impurities in non-controlled products [29,30] can pose several risks to vulnerable populations such as children and adolescents and pregnant and lactating women [31]. This is particularly important regarding the long-term potential deleterious effects of ∆9-THC on neurodevelopment, the associated risk of onset of chronic psychiatric disorders such as anxiety, depression, and psychosis, as well as cognitive impairment and addiction [32,33].

When products are prescribed for clinical conditions without regulatory support regarding their efficacy and safety, and in the absence of proper pharmacovigilance control, GAPs, GMPs, and GLPs, patients may be exposed to interventions with an uncertain risk-benefit balance [8]. This can lead to unpredictable treatment failures, underreported medium- and long-term side effects, potential patient harm, and increased treatment costs [28].

Although some authors argue in favor of the therapeutic use of extracts from the perspective of pharmacological synergy between various phytocannabinoids and non-phytocannabinoid components (described as the “entourage effect”) [34], this hypothesis has proven to be too imprecise and nonspecific to designate the full potential of pharmacological interactions that can occur between these components in cannabis extracts [35,36,37]. Until the methodological and regulatory challenges associated with these products (along with other limitations related to this complex supply chain) are resolved, and evidence provided by RCTs is available [7,8], caution and prudence are necessary on the part of clinicians and stakeholders concerning prescription and regulation.

Therefore, the present review article explores the differences between purified CBD and cannabis extracts enriched with CBD, focusing on their efficacy, safety, and adherence to pharmacological gold standards. It also provides a narrative review of studies that support the rational decision-making of clinicians when prescribing CBD-based products, considering regulation and access concerns. By comparing these two forms, we aim to provide a comprehensive understanding that can guide healthcare providers, researchers, and consumers in making informed decisions about CBD products.

A non-systematic search was conducted in the PubMed, Embase, Web of Science, Scopus, and Lilacs databases to identify relevant studies in the field (including reviews and original studies) that enable a rational and evidence-based discussion.

## 2. Cannabis as Medication and Abuse Drug: Historical View of Market and Regulation Boundaries

*Cannabis sativa* L. (Cannabaceae) has been cultivated for millennia, serving diverse purposes, including fiber, food, religious, recreational, and medicinal uses [38,39,40,41]. While its therapeutic potential was acknowledged in ancient times, significant interest in its medicinal applications in the Western world emerged in the 19th century [40,41], leading to its inclusion in various official pharmacopeias, such as the British Pharmacopeia and the first Brazilian Pharmacopeia, where it was recognized for anti-inflammatory, analgesic, antiemetic, and anticonvulsant uses [8,38,39].

However, due to a lack of knowledge about its active components and inadequate standardization, there was high variability in therapeutic effects. Additionally, the rise of synthetic drugs in the early 20th century and concerns over cannabis abuse resulted in restrictive regulations in multiple countries [16,39]. Following strict classifications by the United Nations Conventions on Narcotic Drug Control (1961, 1971, 1988), cannabis was categorized as a Schedule I and IV substance, indicating significant control [39,42]. Despite this, it remains the most widely consumed illicit drug globally [7]. A comprehensive review of recreational cannabis policies is beyond the scope of this article.

In the past 60 years, following the isolation of key active components [43,44,45], scientific interest in cannabis has resurged, driven by preclinical and clinical research evidencing the therapeutic potential of specific phytocannabinoids [41]. This renewed interest has prompted international authorities to reconsider regulatory restrictions on cannabis for research and medicinal use [16,39]. In 2019, the WHO’s Expert Committee on Drug Dependence recommended removing cannabis from Schedule IV while retaining it in Schedule I, a suggestion adopted by the United Nations Commission on Narcotic Drugs in December 2020 [46,47]. This resolution reflects the UN’s recognition of cannabis’s medicinal potential.

Despite its reclassification, creating evidence-based regulations to address health risks and establish quality standards for cannabis products remains a challenge. As of March 2021, thirty-six countries had enacted regulatory measures for medical cannabis, varying significantly based on sociopolitical conditions—from access to registered cannabinoid medications to medical prescriptions for unregistered products [8]. Clinicians must navigate these complex regulatory frameworks, as they directly influence the safety, efficacy, and quality of cannabis-based products available for patient use.

## 3. Phytocannabinoids, Synthetic Cannabinoids, and Non-Cannabinoid Compounds Derived from *Cannabis* That Hold Therapeutic Potential

Currently, research has identified that, aside from Δ9-THC and CBD, which are well-documented and widely studied, the cannabis plant comprises roughly 565 constituents across 23 classes of compounds. These include more than 129 cannabinoids, 120 terpenoids (comprising 61 monoterpenes, 52 sesquiterpenes, and 5 triterpenoids), 26 flavonoids, 11 steroids, among others [48,49,50].

Perhaps the most well-known class of compounds in cannabis is the phytocannabinoids. These are a group of terpene-phenolic compounds produced through the plant’s secondary metabolism in trichomes of female inflorescences [51], many of which directly modulate the endogenous endocannabinoid system [48]. These naturally occurring cannabinoids are distributed among 10 subclasses, including Δ9-THC and Δ8-THC, cannabidiol (CBD), cannabigerol (CBG), cannabinol (CBN), cannabidinol (CBND), cannabicyclol (CBL), cannabitriol (CBT), cannabielsoin (CBE), and other miscellaneous types (30 known) [52].

In the plant, phytocannabinoids can usually be present in either acidic or neutral forms. The acidic forms are synthesized in the plant via enzymes (e.g., cannabidiolic acid (CBDA) produced by CBDA synthase), while neutral forms are created via decarboxylation from exposure to light or heat [48,49,51,52]. Besides Δ9-THC and CBD, various other phytocannabinoids, including cannabidivarin (CBDV), tetrahydrocannabivarin (THCV), cannabigerol (CBG), cannabichromene (CBC), and cannabinol (CBN), produce pharmacological effects of potential therapeutic relevance [37,51]. Many phytocannabinoids also have chiral centers in their molecular structures [53]. This means there could be one or more additional enantiomers, mirror images with unique three-dimensional shapes, and potentially different physiological effects [54].

It is important to distinguish that, in addition to cannabinoids derived from plants, numerous synthetic cannabinoids have been created and studied. These encompass pharmaceutical grade compounds that resemble the chemical structure of naturally occurring phytocannabinoids found in cannabis plants, like dronabinol (an oral synthetic Δ9-THC formulation) and ZYN002 (a synthetic CBD gel for transdermal application). Most cannabinoids and their derivatives originate from natural sources through the cultivation of cannabis. However, their content can vary significantly due to a range of influences, including soil characteristics, climatic factors, and inherent challenges associated with the extraction and purification of phytocannabinoids of therapeutic interest. Consequently, the interest in obtaining cannabinoids through chemical synthesis has been growing among an increasing number of researchers [55].

However, the notion that cannabinoids are the only components with therapeutic properties in cannabis has been changing in recent years. This is due to increasing scientific interest in investigating the therapeutic contributions of terpenes and flavonoids. Terpenes are lipophilic organic compounds abundant in the plant’s oils, responsible for its characteristic aroma. Their distribution in the cannabis plant can vary substantially depending on the chemovars [56] and on the conditions and duration of drying and storage [57]. Due to their therapeutic properties in other plants [58], terpenes are being investigated as potential contributors to the therapeutic value of the cannabis plant [59]. Also, flavonoids constitute a broad group of bioactive compounds known for providing color and protection against UV radiation. Their role in cannabis is not yet fully understood, and the literature on this topic remains scarce [60].

## 4. Cannabinoid Drug Development

### 4.1. Phytotherapy and the ‘Medical Cannabis’ vs. ‘Single Molecule’ Approach

Given the diverse array of compounds derived from cannabis and their synthetic counterparts that interact with the endocannabinoid system to produce therapeutic effects, two primary paths have emerged over the past decades for cannabinoid drug development. These paths reflect extensions of traditional pharmacological drug development as proper medications versus botanical-derived drug development within the context of alternative and complementary medicine (ACM) [61,62].

The first path involves a strategy of single-molecule purity standards for phytocannabinoids extracted from the plant matrix or synthetic cannabinoids and their analogs [61]. The second path involves a broader strategy of products directly derived from the plant that may contain various phytocannabinoid compounds across a wide spectrum of distribution and concentration and also under different designations, such as “medical cannabis”, “cannabis-based medicines”, or “cannabis-based products” [61,62]. For a comparison, see Table 1.

The standard approval process for medicines in Western medicine follows the model of isolated compounds that have been systematically developed into medications [61]. As will be discussed further, this branch of medication development possesses characteristics that facilitate studies following the principles of evidence-based medicine, which are necessary for approval by regulatory agencies for specific therapeutic indications based on efficacy and safety criteria.

However, this path underwent a significant shift with the legalization of medicinal use of botanical cannabis, initially in some states of the United States and subsequently in other countries worldwide [8]. This is a significant example in medicine where market access to a drug for medical use occurred through legislation rather than formal approval by regulatory agencies, and where the botanical model was preferred over the single-molecule and pharmacological standard model [61].

In this regard, advancements in understanding the plant’s matrix concerning the variability of its active components through the acquisition of different phenotypes via various selection, cultivation, and extraction methods have led to renewed interest in using the plant as raw material for various therapeutic subproducts in recent decades [62,63,64]. Thus, marketed cannabis-based products, encompassing oils and extracts, as well as the inhalation and decoction of specific parts of the plant, have become designated forms of its therapeutic use again, often overlapping with recreational use as permitted in some countries and allowed by some regulations [35].

### 4.2. Critical Evaluation of the ‘Entourage Effect’ and the ‘Medical cannabis’ Concept Through a ‘Single Molecule’ Approach Perspective

In this context, the ‘Entourage Effect Hypothesis’ [65] has gained new contours within the concept of more than a synergistic therapeutic potential in the context of cannabis phytotherapy [63,66,67], and is cited for advocating the therapeutic use of these products based on theorizing their potential benefits [34,63] without, however, emphasizing the importance of controlled studies to evaluate their safety and effectiveness properly [35,37,68].

Some authors have already pointed out the inconsistency of the validity of this hypothesis within the pharmacokinetic and pharmacodynamic frameworks, considering the great difficulty in controlling multiple variables to support, in fact, a hyper-synergistic pharmacological effect between cannabinoids and non-cannabinoids [35,36,37]. This hypothesis also includes several limitations, including the current lack of clarity regarding which compounds contribute to the effect, which pharmacodynamic effects of cannabis are influenced, and whether this phenomenon can be leveraged to enhance cannabinoid therapeutics rather than adverse effects [61,69].

As such, the results obtained under the scope of this hypothesis have been controversial, with inconsistent results in both the few controlled preclinical [70,71,72] and clinical [73] studies that have examined it [74].

Furthermore, while significant progress has been made in recent years in decomposing the active principles of cannabis and their therapeutic potential [21,49,75], scientific literature also provides solid evidence regarding the health risks of cannabis use as a drug of abuse, concerning the risk of developing chronic psychiatric disorders such as depression and anxiety [76], psychosis [77], cognitive impairment, and the risk of car accidents [78], particularly for younger populations [79]. There is also data in the literature showing that exposure of young people to cannabis-based product advertisements increases the risk of cannabis use disorder [80]. Together, these data raise concerns that have not yet been addressed in the literature about how the increased circulation of these products on the market may affect the recreational use and abuse of cannabis [7,81].

Thus, the very concept of “medical cannabis” becomes extremely fragile in terms of an imbalance of risks and benefits [33]. On one hand, it serves the growing billion-dollar market and advertising of these products well, making them attractive to patients and the general public given the argument that if it is natural, it cannot be harmful [82]. On the other hand, it generates methodological inconsistencies at the research level by creating heterogeneity of terms and products [10,26,61], also involving additional concerns related to contamination with heavy metals, mycotoxins, pesticides, and other impurities [27,29,30]. Consequently, research and clinical decision-making become complex, increasing uncertainty about the effectiveness and safety of these treatments for patients and heightening clinicians’ responsibility for outcomes [17].

Combined, these factors may explain, at least in part, the delay in delivering robust and definitive clinical evidence of the safety and efficacy of cannabis-based medicines, as well as in developing interventions with a favorable cost–benefit balance [62]. Additionally, they contribute to the difficulty for clinicians in adequately reconciling and educating their patients’ expectations about these therapies [17]. From a retrospective perspective, this could indicate a similar path to the devaluation and loss of credibility of phytocannabinoid and cannabis-based medicines research as occurred in the nineteenth century [39].

Analogous to cannabis and cannabinoids, other medications have also been isolated from sources that potentially included greater risks to humans, such as acetylsalicylic acid from willow bark, opioids originating from poppy, and bradykinin and captopril derived from the venom of the *Bothrops jararaca* snake. Although the in natura use of these examples may seem less reasonable today in Western medicine, this is due to the development of products with sufficient quality standards to be reproducible and widely tested for their safety and efficacy, thus being considered regulated medications [83].

Having discussed the differences between the terms “medical cannabis”, “cannabis-based medicines”, “cannabis-based products”, and the concept of cannabinoid-based medications, we will proceed with a description of the properties and indications of CBD at a pharmacological purity level.

## 5. CBD: From Isolation to Therapeutic Applications

**Figure 2:**
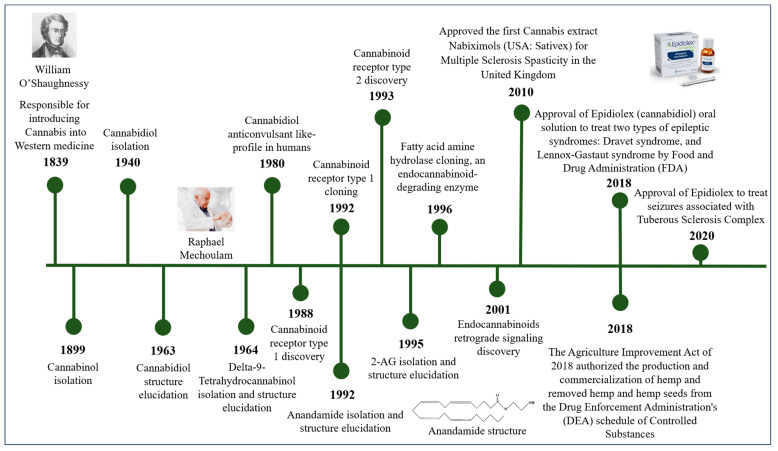
Timeline of the advancement in scientific knowledge on CBD, phytocannabinoids, and the endocannabinoid system, and the development and approval of CBD-based medications.

CBD was first isolated from the cannabis plant in 1940 [43]. For the next two decades, research primarily focused on its isolation, with its precise chemical structure elucidated in 1963 by Israeli researchers led by Raphael Mechoulam [44]. During the 1960s, Mechoulam’s team also clarified the structure and stereochemistry of Δ9-THC and other major cannabinoids [45], setting the stage for subsequent research into their pharmacological properties (see Figure 2).

Until the 1970s, few pharmacological studies on CBD were conducted, suggesting it lacked the psychotomimetic effects associated with Δ9-THC [84]. Early studies from this period indicated that CBD did not mimic cannabis effects, leading to the belief that it was an inactive cannabinoid. This view changed as research revealed significant variations in the effects of different cannabis extracts that could not be explained solely by differing Δ9-THC levels [85,86]. This led to the hypothesis that other cannabinoids, especially CBD, might influence Δ9-THC’s effects.

In this context, a Brazilian team led by Elisaldo Carlini began investigating the effects of CBD and other cannabinoids [87]. Their research suggested that CBD had distinct pharmacological effects, including anticonvulsant properties in animal models [88,89], prompting the first placebo-controlled studies in patients with refractory epilepsy [90,91] and raising the hypothesis of using isolated CBD for the treatment of this condition.

Subsequent research showed that CBD could inhibit or enhance Δ9-THC’s effects in animals, depending on their ratio and dosage [92,93]. Studies on humans found that high oral doses of Δ9-THC induced anxiety and psychotic symptoms, which were mitigated by co-administration of CBD [1].

These findings have been crucial in understanding the varied effects of marijuana across populations and suggest that CBD possesses its own pharmacological properties. Subsequent research has explored these effects extensively, resulting in the current understanding that CBD exhibits a wide range of actions and potential therapeutic uses, particularly in neuropsychiatric conditions [94,95].

Despite the increase in studies on CBD and Δ9-THC in the 1970s, interest in CBD’s therapeutic profile waned in the following decades. However, some groups, particularly the Brazilian teams led by professors Carlini and Zuardi, continued to investigate the anticonvulsant [91,96,97,98], anxiolytic [1,99,100,101] and antipsychotic [102,103] properties of CBD in preclinical and clinical studies [94].

A renewed interest in cannabinoid research emerged after the identification of cannabinoid receptors CB1 and CB2 in the brain and peripheral tissues [104,105] and the discovery of endocannabinoids such as anandamide [106], 2-arachidonoyl-glycerol (2-AG), and its degradation enzymes [107]. These findings suggested the existence of an endocannabinoid system, prompting studies into the pharmacological activities of endocannabinoids as retrograde synaptic messengers [108,109,110], their synthesis on demand from postsynaptic membrane phospholipids [111], and their degradation by the enzymes FAAH (fatty acid amide hydrolase) and MAGL (monoacylglycerol lipase), respectively, in presynaptic neurons [111,112,113,114].

These discoveries led to investigations into how disruptions in the endocannabinoid system could be involved in mental conditions like anxiety and psychotic disorders, and its interaction with other neurobiological processes in the brain [115,116,117,118]. This ushered in a new era of research into the therapeutic properties of cannabis constituents, including CBD’s anxiolytic, antipsychotic, and anticonvulsant effects, through its interactions with the endocannabinoid system and other brain sites [119,120,121].

## 6. Pharmacology of CBD

Most varieties of the cannabis plant produce an unstable compound called cannabidiolic acid (CBDA), which is non-enzymatically decarboxylated with the aid of heat to produce CBD [49,51]. CBD can exist in more than one form. It consists of a terpenophenolic molecule containing 21 carbon atoms with the formula C21H30O2 [122] and two chiral centers, leading to four stereoisomers [123]. The cannabis plant naturally produces one of these, known as (−)-CBD, while its counterpart, (+)-CBD, is not present in *Cannabis sativa*. The (+)-CBD exhibits distinct properties, such as high affinity for CB1 receptors [54].

CBDA can be more potent than CBD in certain situations, but its chemical instability complicates its use [51]. Chemical modifications to enhance its stability have been tested in preclinical studies. Other drugs modified from the CBD molecule have also been synthesized, with many showing therapeutic potential in animal models [124].

### 6.1. Pharmacodynamics

Initially, conducted in vitro studies have identified over 65 potential molecular targets, including enzymes (49%), transporters (20%), receptors (15%), and ion channels (15%), which vary with CBD dosage [125,126,127].

Several mechanisms have been suggested for CBD’s action: activation of TRPV1 receptors, inhibition of anandamide reuptake and metabolism, inhibition of adenosine reuptake, antagonism of GPR55, agonism of PPARγ and 5-HT1A receptors, and increased intracellular Ca++ [128,129,130,131,132,133].

Early research focused on CB1 and CB2 receptors, where Δ9-THC acts as a partial agonist. However, CBD does not bind to the orthostatic binding site of CB1 and CB2 receptors [134,135,136] (see Table 2). Some studies suggest CBD could act as a negative allosteric modulator of CB1 and CB2 receptors [137,138,139]. While this could explain the mitigation of some Δ9-THC effects [140], in vivo results remain debated.

CBD can inhibit the enzyme FAAH (fatty acid amide hydrolase) [141,142,143], which metabolizes anandamide, indirectly increasing this neurotransmitter and interacting with CB receptors, unlike agonists that activate CB1 receptors indiscriminately. Animal models suggest this mechanism may be involved in CBD’s effects on aversive memories, obsessive-compulsive disorder models, and chronic stress consequences [143,144].

Anandamide, a partial agonist of CB1 and CB2 receptors, also activates vanilloid receptors (transient receptor potential cation channel subfamily V member 1 or TRPV1) [145], a subfamily of ion channels involved in inflammation and nociception in the periphery [146] and in glutamate release in the central nervous system [147]. In addition to direct TRPV1 activation, CBD may enhance anandamide’s action on these receptors by inhibiting its metabolism [148]. These effects could be involved in CBD’s inverted U-shaped dose-response curves and antipsychotic effects [149].

Initial in vitro studies suggested CBD could be a 5-HT1A serotonin receptor agonist [150], explaining its broad therapeutic spectrum, given serotonin’s role in various central nervous system pathologies. Subsequent studies showed that some CBD effects, such as the anxiolytic [151], antidepressant [152], anti-stress [153], neuroprotective [154], analgesic [155], and anti-cataleptic [156], are blocked by reversible 5-HT1A antagonists. However, the molecular mechanism by which CBD facilitates 5-HT1A receptor activation remains unclear. Recent research suggests CBD may not act as a direct agonist but likely facilitates 5-HT1A-mediated neurotransmission as a positive allosteric modulator [150,157,158].

Other mechanisms associated with CBD effects include positive allosteric activity on GABA-A receptors [159,160] and GPR55 antagonism [161].

Therefore, the pharmacological complexity of CBD remains a challenge for understanding its effects. While drug development traditionally focuses on creating increasingly selective compounds to enhance therapeutic effects and reduce side effects, CBD’s broad spectrum of potential therapeutic effects likely results from interactions with various molecular targets, exerting fine modulatory actions rather than potent activation or inhibition [162].

### 6.2. Pharmacokinetics of CBD

The observed effects of CBD are influenced by dose, formulation, and route of administration. Like Δ9-THC, CBD is a highly lipophilic molecule, which significantly determines its pharmacokinetic characteristics [163].

#### 6.2.1. Absorption

CBD is commonly administered orally, topically, inhaled, or vaporized [128]. Inhalation allows CBD to be efficiently absorbed into the lungs from the bloodstream, showing pharmacokinetics similar to intravenous administration [164]. Intravenous administration enables CBD to quickly cross the blood-brain barrier, distributing to the brain, adipose tissue, and other organs, although few studies have investigated this pathway [164]. Due to its lipophilicity, CBD can be slowly released from the adipose tissue [2].

When inhaled, CBD reaches peak plasma concentrations (Cmax) within 5–10 min (Tmax). The bioavailability via this route is up to 31% and depends on factors such as inhalation characteristics, the device used for inhalation, particle size, and deposition site within the respiratory system. However, the need for specialized equipment limits the widespread use of these administration methods [165].

Oral administration results in a variable pharmacokinetic profile, likely due to the poor water solubility of CBD [166]. Maximum plasma concentrations achieved through oral intake are lower than those from inhalation, with an oral bioavailability of 6% in humans, reflecting, among other factors, extensive first-pass metabolism. After oral administration, the plasma concentration of its main metabolite is about 40 times higher than that of CBD, and up to 75% of an oral dose of CBD is eliminated by first-pass hepatic metabolism before reaching systemic circulation [167]. The time to reach peak plasma concentration (Tmax) via this route is about 120 min but can vary depending on the dose and other factors [168].

The oral bioavailability of CBD is significantly influenced by factors such as the vehicle used and food intake [168,169]. For example, a recent study observed that when administered dissolved in corn oil instead of purified powder, CBD (150 mg) reached four times higher Cmax [169]. Additionally, CBD’s oral bioavailability is notably affected by food. Being a highly lipophilic molecule, CBD can dissolve in the fats present in food, increasing its oral availability. Indeed, when administered with fatty foods, its availability increases by approximately 3 to 5 times [170,171,172].

Therefore, there is potential to develop enhanced CBD formulations that offer increased oral bioavailability and are less affected by food interactions [167]. Some promising studies have evaluated the impact of self-emulsifying drug delivery formulation technologies in developing formulations that minimize the impact of CBD’s liposolubility on inter- and intra-subject variability in absorption and distribution [173,174].

Despite some in vitro studies suggesting that CBD can convert to Δ9-THC or Δ8-THC in acidic conditions, raising concerns that this process might occur in the stomach, various in vivo studies have indicated that this does not happen in humans under clinical use conditions [175,176].

Administration via oromucosal routes (e.g., sprays like nabiximols) and sublingual routes offers a more consistent pharmacokinetic profile than oral administration [168]. By avoiding extensive first-pass metabolism, these routes achieve plasma concentrations with less variability than those produced by the same dose orally, making them useful in situations requiring rapid effects, such as pain relief [177].

Cannabinoids can also be administered transdermally, with CBD’s skin permeability being about 10 times greater than that of THC. Factors affecting absorption via this route include local blood flow and skin permeability. Given the extreme lipophilicity of these compounds, transdermal administration requires measures to increase skin permeability [178].

#### 6.2.2. Distribution

The distribution of CBD to various tissues, including the central nervous system, can be affected by body composition, such as the higher relative amount of fat in women and the elderly [168]. CBD has a high volume of distribution (Vd) and, with chronic use, can accumulate in adipose tissue [179].

Some animal studies using CBD administration in knockout rats for the expression of P-glycoproteins (P-gp) and breast cancer resistance protein (Bcrp) have shown that CBD is not a substrate for the action of these transporters, meaning they do not reduce the transport of CBD to the nervous system [180]. These proteins are expressed in the blood-brain barrier and act by pumping drugs such as risperidone out of the central nervous system, which has been hypothesized as a mechanism of intrinsic pharmacological resistance [180]. More recent studies have shown interest in investigating whether CBD could inhibit the action of these transporters on other co-administered drugs, potentially modifying a factor of pharmacoresistance for drugs acting on the central nervous system, such as psychotropic and anticonvulsant drugs [181].

#### 6.2.3. Metabolism

CBD is metabolized in the liver by cytochrome P450 enzymes, including CYP2C19, CYP3A4, CYP1A1, CYP1A2, CYP2C9, and CYP2D6, and is converted into 7-hydroxycannabidiol (7-OH-CBD) [182]. After hydroxylation, CBD undergoes further metabolism in the liver before being excreted primarily through feces and urine [165]. Preclinical and clinical studies suggest that 7-OH-CBD is an active metabolite, also exerting anticonvulsant activity [183,184].

CBD has a half-life of 18–32 h and a clearance rate of 57.6–93.6 L/h [165]. Recent studies propose that CBD undergoes multiphasic elimination, with an initial rapid phase and a slower terminal phase, likely reflecting its slow absorption and distribution to tissues [168].

CBD is well tolerated at therapeutic doses and has a good safety profile [4,185]. However, some studies have indicated that CBD has potent inhibitory effects on CYP2C, CYP2D6, and CYP3A isoforms [186], which can interfere with other drugs metabolized by these isoenzymes (see further discussion on potential drug–drug interactions).

### 6.3. CBD in Special Populations

Most studies investigating the pharmacokinetic properties of CBD and other cannabinoids have been conducted in adults, predominantly men. The number of pharmacokinetic studies in children and the elderly is limited. Additionally, there is a lack of studies on the prolonged use of oral CBD in patients with renal and hepatic insufficiency [187]. Given the previously mentioned high degree of hepatic clearance experienced by orally administered CBD, this latter population warrants additional attention and caution.

Age-related changes in drug metabolism, such as aging, pregnancy, decreased hepatic and renal function, can alter the pharmacokinetics of CBD [187]. The aged population is at higher risk for drug–drug interactions due to polypharmacy, and the potential for increased sensitivity to CBD’s effects needs further investigation.

A meta-analysis evaluating the efficacy and safety of medical cannabinoids in eight RCTs reinforced the findings that only purified CBD demonstrated accumulated efficacy in the pediatric population for the therapeutic outcome of controlling epileptic seizures in Dravet syndrome [188]. It indicated that all groups of cannabinoids (including purified CBD, nabilone, nabiximols, and standardized Δ9-THC:CBD extract 1:20) were associated with severe adverse effects, including mental adverse events [188]. These safety findings are significant and align with case reports indicating severe neurobehavioral side effects in children with cannabis intoxication, such as ataxia and prolonged coma [189,190,191,192].

Children and adolescents may be affected by cannabinoids differently than adults, considering the differences in neurodevelopment and their pharmacodynamic and pharmacokinetic implications for safety [193]. As long-term exposure to cannabinoids may pose risks to neurodevelopment, the use of CBD and other cannabinoids in the pediatric population requires extreme caution in weighing risks and benefits.

## 7. Clinical Profile of Pharmaceutical Grade CBD

### 7.1. Adverse Effects

Recent reviews indicate that the acute, sub-chronic, and chronic administration of CBD in various doses, especially in oral formulations, generally results in mild to moderate side effects, including gastrointestinal symptoms, drowsiness, loss of appetite, and elevated liver enzymes [181,185]. Serious adverse effects are rare, with hypertransaminasemia, marked by serum levels exceeding three times the upper limit, being the most notable. Other reported complications involve seizures, sedation, lethargy, upper respiratory tract infections, and rashes. These adverse events are mainly associated with doses higher than those recommended for human therapy and concurrent use of antiepileptic drugs such as valproate [181,194,195]. The long-term effects of CBD, however, are still unknown [196], demanding caution regarding its use in children, adolescents, and during pregnancy and lactation [31].

### 7.2. CBD–Drug Interactions

A drug interaction involves a quantifiable modification in the magnitude or duration of effects due to the concurrent or prior administration of other drugs, food, or the patient’s pathophysiologic conditions [197]. These interactions can be additive (1 + 1 = 2), synergistic (1 + 1 > 2), or antagonistic (1 + 1 < 2). Typically, most interactions documented in the literature are attributed to pharmacokinetic mechanisms, involving alterations in the activity of cytochrome P450 (CYP) isoenzymes, P-glycoprotein (P-gp), or other drug transporters [198].

Given that CBD inhibits cytochrome P450 (notably the isoforms 2C19 and 3A4) and UDP-glucuronyltransferase isoforms, it is crucial to assess potential drug–drug interactions carefully [4,167], considering the metabolism of other drugs co-administered with CBD [199]. Also, drug–drug interactions affecting CBD metabolism are likely to have a prominent impact on its systemic exposure after oral administration [200].

Thus, the interaction of CBD with drugs metabolized via the cytochrome P450 pathway can involve bidirectional interference, with CBD and other drugs potentially altering each other’s serum levels. This is particularly relevant for the CYP3A4 isoform, as approximately 60% of clinically used drugs are metabolized by this pathway [167]. These interactions could theoretically result in increased side effects and decreased therapeutic effects over time, underscoring the importance of controlled studies in evaluating the safety of CBD’s pharmacological interactions and their implications for clinical outcomes [167].

Studies on drugs that significantly interfere with the CYP3A4 pathway have shown an increase in CBD plasma concentration in the presence of a CYP3A4 inhibitor (ketoconazole), leading to significant side effects [201,202]. Similarly, the administration of CYP3A4 inducers (rifampicin) reduced CBD serum levels [201,202]. Although in vitro studies suggest that CYP2C19 plays an important role in CBD metabolism, its concentrations did not change in the presence of potent inhibitors of this enzyme.

Additionally, CBD also affects other CYP450 isoenzymes, and interactions may occur in different metabolic pathways, such as 5′-diphospho-glucuronosyltransferase (UGT), as well as interactions at the level of transporters and receptors [181,198].

Among the various potential drug interactions when CBD is used, those with anticonvulsants, benzodiazepines, antidepressants, anticoagulants, and opioids have shown significant implications in clinical practice.

#### 7.2.1. Anticonvulsants

Among anticonvulsants, CBD interacts bidirectionally with clobazam, potentially via the CYP2C19 enzyme [203,204,205]. CBD significantly increases the plasma concentration of clobazam and its active metabolite, N-desmethylclobazam, reducing its clearance by up to five times, while clobazam induces an increase in the 7-hydroxy CBD metabolite [204,205]. Although the antiepileptic action of CBD is independent of this interaction with clobazam, patients using both medications concomitantly may experience better seizure control compared to those using either drug alone. Conversely, there may also be a higher incidence of side effects such as sedation, ataxia, cognitive impairments, cough, difficulty breathing, fever, and behavioral changes. Rigorous monitoring of side effects is recommended during the concomitant use of both medications, including serum clobazam level monitoring if available [198].

In human studies, the concomitant use of CBD and valproic acid is associated with hepatotoxicity, indicated by altered liver enzymes (transaminases and gamma-glutamyl transferase) and thrombocytopenia in pediatric patients [203,206]. These effects tend to be dose-dependent and resolve with the reduction or discontinuation of one of the drugs, although rare cases of severe liver injury leading to jaundice have been reported [203,206]. It is advisable to conduct laboratory evaluations to monitor hepatic profiles and valproic acid levels before introducing CBD into the prescription, with follow-up tests at six weeks.

Moreover, preliminary studies suggest that CBD may interact with other anticonvulsants, potentially increasing the plasma concentration of gabapentin, oxcarbazepine, and topiramate [203]. No interactions have been reported with pregabalin and levetiracetam [203]. Overall, the results are mixed and preliminary, not seeming to bring significant changes in the clinical outcomes of patients, but clinicians should always be vigilant for worsening side effects during the combined use of CBD with these anticonvulsants [198,203].

#### 7.2.2. Benzodiazepines and Antidepressants

In vitro studies suggest that CBD exhibits pharmacokinetic interaction with citalopram and escitalopram through probable CYP3A4 and CYP2C19 interactions, increasing the plasma concentration of these drugs by reducing their metabolism [207]. This interaction does not appear to result in serious side effects. Similarly, preliminary evidence suggests that CBD may interact with sertraline, fluoxetine, and mirtazapine. There is still insufficient evidence regarding the potential interaction of CBD with tricyclic antidepressants, drugs known to increase the QT interval and share the same metabolic pathways as CBD [198].

As mentioned in the case of clobazam, CBD’s action on CYP3A4 and CYP2C19 can interfere with the metabolism of other benzodiazepines such as diazepam and midazolam [198].

#### 7.2.3. Anticoagulants

Case reports suggest that CBD may increase the efficacy of warfarin [208], resulting in gastrointestinal bleeding, which has also been observed during the recreational use of cannabis [209,210,211]. Since other oral anticoagulants, such as rivaroxaban, share the same metabolic pathways, similar interactions are possible, although no human cases have been reported yet. Therefore, it is advisable to be vigilant about these potential interactions, monitoring coagulation tests, and considering dose adjustments of warfarin if necessary [198].

#### 7.2.4. Opioids

Some case reports indicate that CBD may increase the plasma concentration of opioids such as buprenorphine [212] and methadone [213] by inhibiting their metabolism through the CYP3A4 and CYP2C19 isoforms. It remains uncertain whether these effects could be associated with increased analgesic potency of opioids, facilitate their discontinuation in cases of dependence, or result in increased side effects.

It is important to emphasize that, based on available evidence, CBD is generally considered a relatively safe medication, with few clinically significant drug interactions and no absolute contraindications. However, evidence on the pharmacological interactions of cannabis and its derived products remains limited [214]. Many implicated interactions are still theoretical or demonstrated in small studies [198]. Therefore, following the principle of non-maleficence, it is advisable to exercise caution when considering potential pharmacological interactions related to cannabis-derived products such as extracts and whole plants until more robust evidence is available.

### 7.3. CBD’s Bell-Shaped Curve Dose-Response

Understanding the dose-response profile is an important pharmacological factor in drug studies, allowing correlation with effectiveness data for a specific clinical condition. To date, CBD has shown an established effective dose range only for the treatment of refractory childhood epilepsy in Dravet and Lennox–Gastaut syndromes, for which it has received regulatory approval as a medication [215].

However, preliminary academic studies involving translational research that correlates preclinical models and human studies suggest that some therapeutic effects of CBD, notably its anxiolytic effect, follow an “inverted U” dose-response pattern [3]. While some pioneering studies [93] reported that CBD (10 mg/kg) could reduce conditioned emotional responses in rats, others did not observe any effects of CBD (100 mg/kg) [3]. These apparent contradictions were reviewed by Guimarães [100,216] using an anxiety-sensitive test—the elevated plus maze. Using this model and conducting a comprehensive dose-response analysis, they demonstrated that CBD induces anxiolytic effects at lower doses (2.5–10 mg/kg) in rats, which completely disappear at higher doses [100]. Subsequently, a study involving healthy volunteers subjected to a validated experimental anxiety model (Public Speaking Simulation Test) demonstrated that, among a broad range of single doses studied (150 mg, 300 mg, and 600 mg), CBD exerted the most effective acute anxiolytic effect at the intermediate dose of 300 mg [217]. These results were replicated and expanded in a later study that employed the real public speaking test model in a range of single doses involving 100 mg, 300 mg, and 900 mg, again with the 300 mg dose showing superior acute anxiolytic effect compared to the others [218]. These findings support the idea of an inverted U-shaped dose-response effect for CBD, which presumably involves different dose profiles depending on the therapeutic indication [3].

Indeed, in addition to anxiety models [219], some studies involving animal models for pain [220] and vomiting [157] have also reported inverted U-shaped dose-response effects. However, this hypothesis has not been directly studied in other clinical conditions, highlighting the need to delineate an accurate therapeutic range for each clinical condition [3].

It is also important to note that this relationship has been well-established for the acute anxiolytic effect of single doses of CBD in preliminary social anxiety model studies involving healthy volunteers, not involving the determination of an effective therapeutic range for the treatment of anxiety disorders, which can only be supported by placebo-controlled clinical trials [221,222]. Notably, these studies are still lacking for many clinical conditions for which CBD is recommended [223].

The exception, again, is the indication of CBD in treatment-resistant childhood epilepsies, such as Dravet and Lennox–Gastaut syndromes [215]. In these cases, the recommended starting dose is 5 mg/kg/day with increments every two weeks, reaching up to 25 mg/kg/day if tolerated [18,224,225]. The suggested starting dose for adults is 200 mg/day, increasing to 1.500 mg/day, with the most frequent dose being around 800 mg/day [226].

An important fact is that CBD in pharmacological purity is an expensive product, and titrating to high doses suggested in definitive studies involves a significant investment in treatment, often limiting access for many patients [8]. Due to this fact, some authors have argued for the “entourage effect” and the combination of cannabinoids as a way to overcome the inverted U-shaped dose-response effect of CBD, aiming to use CBD-enriched extracts at lower doses to achieve therapeutic efficacy [63,227,228].

Although preliminary studies involving pain models in animals suggested the need for lower doses of CBD for pain control using extracts with Δ9-THC (a condition for which the combination of both cannabinoids seems to be more effective than either alone) [227], these results should be viewed with caution and should not be generalized to other conditions without properly controlled studies [37]. Furthermore, as discussed later, the combination of cannabinoids may not necessarily result in therapeutic effects for all clinical indications.

## 8. CBD and Δ9-THC

The pharmacology of Δ9-THC, although well-known, is complex. Δ9-THC interacts with various molecular targets and acts as a partial agonist at CB1 and CB2 receptors, primarily inhibiting presynaptic neurons via CB1 receptors. Chronic administration of Δ9-THC can lead to tolerance of its effects and the action of endocannabinoids [229,230].

In animal models, Δ9-THC and its analogs produce the so-called cannabinoid tetrad (hypolocomotion, catalepsy, analgesia, and hypothermia) [231]. In humans, Δ9-THC is most commonly associated with the psychotomimetic effects of cannabis use, including dysphoria, anxiety, sedation, psychotic symptoms, and psychomotor impairment [232]. More recently, its therapeutic potentials, including analgesic, muscle relaxant, antispasmodic, and anti-inflammatory properties, have been recognized [75].

Clinical emphasis may be placed on the analgesic effect, which, although still under investigation, likely correlates with higher serum levels of Δ9-THC and, consequently, with its potential for intoxication [233,234,235]. In clinical trials assessing the analgesic efficacy of cannabis, Δ9-THC concentrations were close to <10% [234,236,237,238]. In fact, significantly lower concentrations of Δ9-THC (1–3%) were used in some studies and did not result in clinical efficacy [234,238]. Furthermore, adverse effects and treatment discontinuation seemingly tend to increase with higher concentrations used in these studies. This correlation between Δ9-THC concentration and the potential for intoxication and adverse effects has become an increasing concern as the availability of products with high levels of Δ9-THC and cannabis potency rises [239,240].

A more debated issue is whether and how CBD interacts with Δ9-THC when administered together. Many researchers have suggested that CBD may influence the effects of Δ9-THC, enhancing its clinical efficacy and reducing side effects [61,241]. The main evidence for this hypothesis comes from naturalistic studies indicating that cannabis with high CBD content is associated with lower rates of cognitive impairment and verbal memory deficits [242,243,244]. Additionally, pioneering human studies have shown that when co-administered with Δ9-THC, CBD minimizes the occurrence of intense psychoactive effects such as anxiety and psychosis [1].

Evidence from repeated dosing studies is more varied. A parallel group randomized controlled trial (*n* = 177) in patients with intractable cancer-related pain showed that a combination of Δ9-THC (2.7 mg) and CBD (2.5 mg) in an oromucosal spray produced a significant improvement on a pain rating scale compared to placebo, whereas the Δ9-THC (2.7 mg) group showed no significant change. Twice as many patients (43%) taking Δ9-THC and CBD showed a 30% pain reduction (on a 0–10 Numerical Rating Scale) from baseline compared to placebo (21% of patients) [245]. However, other studies have found Δ9-THC alone to be more clinically effective than the combination of the two in chronic pain, fibromyalgia, and neuropathic pain [235,246,247].

Much uncertainty also exists regarding whether CBD alters the pharmacokinetic profile of Δ9-THC, due to the great heterogeneity of doses and administration routes used in studies evaluating these hypotheses [69].

Among approved medications by regulatory agencies, Sativex is an oromucosal spray formulation containing a 1:1 ratio of CBD and Δ9-THC, shown to be effective as an add-on medication in the treatment of refractory spasticity and pain in multiple sclerosis [248]. It is likely that Δ9-THC:CBD ratios greater than 1:1 tend to exhibit intoxicating pharmacological effects and are associated with increased side effects linked to Δ9-THC, whereas Δ9-THC:CBD ratios less than 1:6 may mitigate these effects [14]; however, this remains an arbitrary categorization. A comprehensive review of the literature that considers the confounding variables involving the ratios of combinations of these cannabinoids, routes of administration, and types of formulations and preparations is necessary. A systematic review is being conducted to assess studies that investigated oral Δ9-THC pharmacokinetics in different cannabinoid formulations (CRD42024569571). Despite this, there are no consistent results on the dose ranges of these cannabinoids in which CBD modulates the therapeutic effects of Δ9-THC to enhance its efficacy and reduce side effects [69].

Although this hypothesis carries positive expectations, caution is required when translating it into clinical practice (considering that Δ9-THC has greater pharmacological potency than CBD), and more controlled studies are needed to evaluate the efficacy and safety of appropriate and standardized combinations for different clinical indications [69].

The literature on the interaction of CBD with other phytocannabinoids and non-cannabinoid compounds is still in its infancy, and little is known beyond theoretical speculation about the extent to which these compounds may positively or negatively influence therapeutic properties. See [36,37] for a more in-depth review.

## 9. CBD Content in Commercialized Products

With the legalization of hemp for industrial and medicinal purposes, several countries have seen an increase in the availability of hemp-derived products, particularly CBD products. The lack of regulatory oversight in this industry has led to the availability of CBD products with questionable compound quantities and quality [26].

Several studies conducted in different countries, including the United States [9,26,27,249], the United Kingdom [13], Australia [30], the Netherlands [250], Italy [251], and South Africa [15,30], have reported discrepancies between the information on CBD product labels and their actual chemical content. These discrepancies include significant variations in the actual amount of CBD (both higher and lower than stated) [13,27,29], the presence of other controlled phytocannabinoids (such as Δ9-THC, Δ8-THC, and CBN) in amounts exceeding legal limits [13,27], and the presence of residual solvents in quantities unsuitable for human consumption [30].

These reported irregularities can have various negative implications for the health of patients consuming these products. Firstly, unexpected variations in CBD content can result in subtherapeutic or supratherapeutic doses, potentially leading to treatment failures or increased side effects, respectively [26].

Secondly, undisclosed contamination with Δ9-THC in some products can lead to acute side effects and accidental intoxication due to the presence of this phytocannabinoid, a concern increasingly reported in studies involving both adult and pediatric populations [28,252]. Additionally, the long-term effects of inadvertent Δ9-THC exposure, particularly in pediatric populations undergoing neurodevelopment, could lead to an unexpected increase in psychiatric disorders and cognitive impairments [28,232]. This is particularly relevant as Δ9-THC, a partial agonist of CB1 receptors, has greater pharmacological potency than CBD, correlating with its potential adverse effects [187]. For instance, a CBD product labeled as Δ9-THC-free but found to contain approximately 2.071 mg of Δ9-THC per mL, when taken at doses of 1 mL three times a day, would result in a daily intake of about 6.213 mg of Δ9-THC. For comparison, the recommended starting dose for Dronabinol (synthetic oral Δ9-THC) is 2.5 mg [27]. Additionally, the inadvertent Δ9-THC contamination in CBD products raises potential legal issues such as impaired driving, job loss, child custody issues, and positive doping tests in sports, given the possibility of positive results in sensitive toxicology tests for Δ9-THC [27].

Still in this sense, a review of 8534 herbal products available in the cannabis market for both recreational and medicinal use across nine U.S. states revealed that about 58.5% did not indicate any amount of CBD on their labels. Moreover, when categorized based on the potency of these products to cause intoxication or side effects based on the Δ9-THC:CBD ratio, most medicinal products fall into the potentially intoxicating category (Δ9-THC:CBD > 1). The categories identified as less intoxicating (1:>2 < 6 and ≤1:6 THC:CBD) exhibited the lowest number of products [14].

Thirdly, inadvertent exposure to residual solvents, heavy metals, pesticides, and microorganisms can also have long-term negative effects, particularly in populations with clinical conditions that include immunosuppression [27,30].

Some authors argue that these irregularities partly result from the flexibility of the 2018 Agriculture Improvement Act regarding the regulation of industrial and medicinal hemp-based products (cannabis with <0.3% Δ9-THC by dry weight) in the USA. This act specifies control and oversight only for the Δ9-THC limit in the raw plant material, not the final product (up to the individual states), potentially resulting in products with higher than permitted Δ9-THC concentrations that are not adequately supervised [15,27]. Although some cannabis-derived products, such as broad-spectrum extracts (containing CBD, phytocannabinoids, terpenoids but without Δ9-THC) and full-spectrum extracts (including Δ9-THC < 0.3%), claim differences in their Δ9-THC content to varying degrees, the lack of adequate supervision and oversight of these products raises questions about their quality and actual component quantities [27].

In the Brazilian context, for example, these data are relevant following ANVISA’s (local regulatory agency) Collegiate Board Resolution No. 327/2019. Considering the high demand for access to medicinal cannabinoid-derived products, this resolution allows the circulation of imported products in the national market. Furthermore, some companies have received a five-year sanitary authorization for producing and commercializing cannabinoid products, during which they must prove their efficacy and safety [8]. The examples cited from other countries highlight the need for adequate agency oversight regarding product quality, considering the possibility of label discrepancies, and the need for controlled clinical trials to confirm clinical efficacy.

## 10. Recommendations for Further Research on CBD

Several recent literature reviews on the therapeutic potential of CBD highlight the well-documented lack of placebo-controlled clinical trials needed to provide more robust evidence of its clinical efficacy in different yet nonapproved conditions [7,33,177,223]. These studies become even more essential when evaluating the clinical efficacy of CBD, given the results of recent studies where the placebo effect, associated with the expectation of treatment with CBD for indications such as anxiety and pain [253,254], significantly influenced clinical outcomes [255].

The expectancy effects of treatment can be presumably modulated by various factors, such as the context of access to medication, the regulatory status of the cannabis plant, its therapeutic efficacy in refractory and difficult cases of childhood epilepsy, and media portrayal of it as a natural-derived medication with multiple indications. A recent systematic review indicated that significant attention from social media can elevate expectations and shape the placebo response in future trials. This has the potential to affect clinical trial outcomes, regulatory decisions, clinical practice, and ultimately, patient access to cannabinoids [255].

Although the objective contribution of other factors to the placebo effect has not been rigorously assessed in studies, they may exert significant influence when considering selection and population biases, particularly in open-label and nonrandomized studies [256].

Furthermore, considering the pharmacological profile of CBD, it is crucial to explore the therapeutic dose ranges for specific clinical conditions and potential pharmacological interactions with first-line drugs generally prescribed for these conditions [3]. Given the known interaction between food and the oral administration of CBD, the development of new formulations that minimize these inter- and intrapatient variations is also desirable [167].

Moreover, a significant and challenging confounding factor is the interference of concurrent recreational cannabis use and its influence on the therapeutic response to CBD [7]. This is particularly relevant when considering the potential alteration of the endocannabinoid system tone in patients with acute or chronic cannabis exposure, making the assessment of quantitative phytocannabinoid composition even more complex, even in regulated use scenarios. Added to this complexity is the increasing overlap between sectors of the population reporting both medical and recreational cannabis use [7].

This task is further complicated by the widespread use of varied and often nonspecific terms in the clinical setting, such as “medical cannabis”, and the presence of products on the market that do not meet good manufacturing and labeling standards [13,25,26,35]. Despite these challenges, the use of validated scales that adequately quantify recreational cannabis use and its inclusion in randomized, placebo-controlled studies can help elucidate this issue and reduce the interference of significant confounding factors, aiding complex clinical decision-making [7].

Furthermore, there is a need for more controlled clinical trials that explore the therapeutic potential of standardized cannabis extracts under GAP and GMP and evaluate the impact of different Δ9-THC:CBD ratios on therapeutic outcomes balanced with intoxication effects and other adverse effects associated with Δ9-THC [14].

In this regard, considerations for advancing future research include appropriate regulation linked to dialogue with civil society, patient associations, and related healthcare professionals [8].

## 11. Conclusions

This narrative review summarized the main factors related to research and clinical practice involving the use of CBD, contextualized within a broader framework of regulation and perspectives on the development of new drug therapies. Conceptual clarification is valuable given the diversity of terms currently found in the literature, which often originate from discussions outside the clinical setting and can significantly influence clinical and research decisions [255]. CBD is neither a “silver bullet” nor a panacea. Its therapeutic efficacy at a pharmacological grade of purity has been demonstrated for a still-limited set of conditions. Current evidence of its pharmacological profile and therapeutic potential highlights the need for more controlled studies that can expand the range of conditions for which it has proven clinical efficacy.

Additionally, it is important to highlight the differences between pharmaceutical grade CBD and CBD-enriched cannabis extracts in terms of their composition, particularly regarding the presence of other cannabinoids in the latter group. This distinction is significant concerning Δ9-THC potential acute and chronic adverse effects associated with, or independent of, intoxication, underscoring the need for more controlled studies using standardized extracts under GAP, GMP, and GLPs. Such research is necessary to explore potential balances between Δ9-THC and CBD doses and ratios that enable therapeutic application considering intoxication and adverse effects. This research is important because, even if some patients may enjoy the psychotomimetic effects or are willing to assume the risks of high doses or chronic Δ9-THC consumption, this is not recommended from a medical standpoint.

*Cannabis*, even though approved as ‘medicinal marijuana’ by Canada and Uruguay, and several other states in the United States, without sufficient clinical evidence, cannot be considered a therapeutic because neither the US FDA nor any other regulatory body has approved it as medicine. Based on sufficient clinical data from several clinical trials, CBD is approved for treating two rare forms of epilepsy (Lennox–Gastaut and Dravet syndromes) in young children, but not in adults, and another rare form of brain tumor, tuberous sclerosis complex. CBD in a 1:1 combination with THC (nabiximols, Sativex) is available in many countries except the US, for treating MS-related neuropathic pain. Two other cannabinoid-based medications, synthetic THC (Marinol) and a THC analog (Nabilone), are approved for treating chemotherapy-associated nausea and vomiting and appetite stimulation in AIDS-related wasting syndrome.

Just like any other medication in clinical practice, there is off-label use and compassionate use for the cannabinoid-derived medications described above; however, using these medications outside the conditions specified in the package insert exposes patients to uncertain clinical outcomes.

Neither cannabis nor any other cannabinoid isolated or in combination is approved as a therapeutic, even though these are widely promoted as therapeutics. It is also important that clinicians who deal with patients asking for recommendations of cannabis or CBD for self-treatment must educate their patients about the dangers of using recreational cannabis, which is currently highly potent and is associated with serious adverse health consequences, including cannabis use disorder and psychosis, and that CBD is approved only for treating epilepsy and no other indication.

To this end, the steps for its approval as a medication must be followed so that patients can genuinely benefit from its potential clinical efficacy, if appropriately demonstrated. Researchers and clinicians must remain aware of the complexity of the different interfaces of the topic globally while maintaining their focus on their role in developing best practices for prescription and research based on evidence-based medicine.

## Figures and Tables

**Table 1:** Comparison of pharmaceutical grade cannabidiol, non-pharmaceutical grade cannabidiol, and cannabidiol extracts.

Aspect	Pharmaceutical Grade Cannabidiol	Non-Pharmaceutical Grade Cannabidiol	Cannabidiol Extracts
Purity	99.6–99.9% pure	Variable, typically 70–90%, may contain THC	Variable, contains other cannabinoids and terpenes, including THC
Quality Control	Strict regulatory standards (GMP)	Inconsistent, lacks standardized quality	Variable, often lacks rigorous testing
Label	Correct label and actual CBD composition	Frequent discrepancies between label claims and actual cannabinoid compositions	Frequent discrepancies between label claims and actual cannabinoid compositions
Consistency	High batch-to-batch consistency	Variable	Variable
Safety Profile	Well-documented	Less documented	Not documented
Contaminants	No contaminants, heavily tested	Potential presence of contaminants	Potential presence of contaminants
Legal Status	Legal in most countries with prescription	Legal status varies by region	Legal status varies by region
Usage	Medical and clinical use	Often used for supplements and wellness	Often used for supplements and wellness
Efficacy	High efficacy supported by studies	Efficacy varies, less research available	Efficacy varies, less research available
Cost	High due to quality and testing	Lower to moderate cost	Lower cost
Example Products	Epidiolex^®^, Sativex^®^	Various over-the-counter products	Full-spectrum CBD oils

**Table 2:** Comparative pharmacodynamics of CBD and other phytocannabinoids.

Phytocannabinoid	Pharmacological Targets	Properties
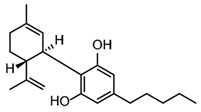 **Cannabidiol (CBD)** **C_21_H_30_O_2_**	- CB_1_ negative allosteric modulator - CB_2_ inverse agonist - 5-HT_1A_ partial agonist - D_2_ partial agonist - Fatty acid amide hydrolase inhibitor - PPAR_γ_ agonist - TRPV_1_ agonist - GPR_55_ antagonist	- Non-psychotomimetic - Anxyolitic - Anticonvulsant - Analgesic - Antipsychotic - Anti-inflammatory - Neuroprotective - Antioxidant - Antibacterial
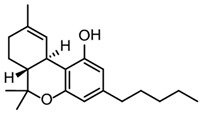 **Delta-9-** **Tetrahydrocannabinol (THC)** **C_21_H_30_O_2_**	- CB_1_ partial agonist - CB_2_ partial agonist - GPR_55_ agonist - PPAR_γ_ agonist - TRPV_2_ agonist - TRPV_3_ agonist - TRPV_4_ agonist	- Psychotomimetic - Stimulation of appetite - Analgesic - Anti-inflammatory - Intoxicant - Antiemetic - Antimicrobial
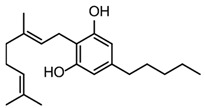 **Cannabigerol (CBG)** **C_21_H_32_O_2_**	- CB_1_ weak agonist - CB_2_ partial agonist - 5-HT_1A_ antagonist - Alpha_2_-adrenergic agonist - PPAR_γ_ agonist - TRPV_1_ agonist	- Non-psychotomimetic - Anti-inflammatory - Analgesic - Neuroprotective - Anxyolitic - Antioxidant - Antibacterial - Antitumor
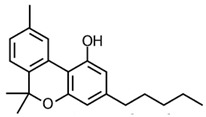 **Cannabinol (CBN)** **C_21_H_26_O_2_**	- CB_1_ partial agonist - CB_2_ partial agonist - TRPV1 agonist - TRPV2 agonist - TRPV3 agonist - TRPV4 agonist	- Psychotomimetic - (weaker than THC) - Stimulation of appetite - Analgesic - Anti-inflammatory - Intoxicant - Sedative - Sleep improvement - Immunosupressant
